# Meditation and the Brain: Attention, Control and Emotion**

**DOI:** 10.4103/0973-1229.77444

**Published:** 2011

**Authors:** Gabriel José Corrêa Mograbi

**Affiliations:** * *Professor of Philosophy of Science, Philosophy of Mind and Epistemology, Federal University of Mato Grosso, Brazil*; ** *Revised and peer reviewed version of a paper read at an International Seminar on Mind, Brain, and Consciousness, Thane College Campus, Thane, India, January 13–15, 2010.*

**Keywords:** *Attention*, *Concentration*, *Emotion*, *Compassion*, *Neural correlates of meditative states*

## Abstract

Meditation has been for long time avoided as a scientific theme because of its complexity and its religious connotations. Fortunately, in the last years, it has increasingly been studied within different neuroscientific experimental protocols. Attention and concentration are surely among the most important topics in these experiments. Notwithstanding this, inhibition of emotions and discursive thoughts are equally important to understand what is at stake during those types of mental processes. I philosophically and technically analyse and compare results from neuroimaging studies, produced by leading authorities on the theme, dealing with two types of meditation: “one-pointed concentration” and “compassion meditation”. Analysing “one-pointed concentration”, I show the differences between novice and expert meditation practitioners in terms of brain activity and connectivity, considering the relationship among increased attention and concentration and decreased activity in areas related to discursive thought and emotion. Analysing “compassion meditation”, I show the importance of the limbic circuitry in emotion sharing. I follow the same strategy of comparing novice and expert meditation practitioners. The conclusion establishes a common structure to those different ways of dealing with emotion during meditation.

## Introduction

For a long time, the scientific literature was not very dedicated to meditation as an empirical issue, maybe considering that the religious or mystic connotations of meditation would jeopardise the seriousness and scientific validity of empirical results, or else because it was considered a form of practice not interesting for the western paradigm of science. Notwithstanding, several seminal and courageous articles and books between the early 60s and late 90s have addressed the issue with relevant and different approaches (Deikman, 1963; Fischer, 1971; Davidson *et al*., 1976; Davidson and Goleman, 1977; Kabat-Zinn *et al*., 1985; West, 1987; Austin, 1999). These seminal works have their undeniable scientific merits as they paved the way for a more mature and detailed empirical understanding of such intricate issues with the development of functional brain imaging techniques.

Fortunately, meditation has being an intriguing source of discussion in the recent literature of neuroscience and appears to be an insightful source of new data about consciousness and brain functioning as can be seen in several recent publications (Lutz *et al*., 2004; Berger *et al*., 2007; Slagter *et al*., 2007; Lutz *et al*., 2007; Grant *et al*., 2010; Goldin and Gross, 2010).

In this article, I concentrate my analysis on two papers that approach two different types of meditations. The former (Brefczynski-Lewis *et al*., 2007) deals with “one-pointed concentration” and the latter (Lutz *et al*., 2008) is a study on “compassion meditation”.

I initially analyse an assertion of one of the authors I am considering: “Meditation refers to a family of mental training practices that are designed to familiarize the practitioner with specific types of mental processes” (Brefczynski-Lewis *et al*., 2007, p11483). First of all, this very general definition of meditation is perfectly in accordance with a materialist and scientific worldview. There is no underlying trace of religiosity or mysticism here. Thus, even though a very general definition, it allows us to depart from a definition not compromised with any claim that would not be possible to be scientifically addressed.

Certainly, we have several different types of meditation and this idea is already supposed as part of the above-cited assertion: meditation is a *family* of mental training and mental processes. I only address two types of meditations here: “one-pointed concentration” and “compassion meditation”. The first type of meditation (my main focus in this writing) is a mental process where the subject concentrates her/his attention on a small object or on her/his own breath not being distracted by concurrent stimuli. The second type of meditation I address here is “compassion meditation”. This type of mental process is based on training by reflection to dismiss the traditional self-centered way of dealing with emotions, and to augment empathy strategies shifting one’s perspective from the first-person to an inter-subjective way of dealing with emotions.

## One-Point Concentration Meditation as a Subject Matter in the Lab

As the authors assert, “The technical term for this meditation in Tibetan literally means one-pointed concentration” (Brefczynski-Lewis *et al*., 2007, p. 11483). The main technique of this type of mental training called “one-pointed concentration” is to focus on a small object and maintain the focused attention without surrendering to concurrent stimuli, monitoring one’s mental activity in such a fashion that sleepiness, agitation, dullness or inner chatter are all avoided. We must highlight something mentioned by the authors – this type of meditation is one of the “most basic”. I would say the simplest and, in some of the eastern traditions, is taken to be a form of initiation to meditation. On the one hand, some critics could take this simplicity as a flaw of the experiment as it does not show what happens in the brain of meditators in more advanced forms of meditation. Probably, people with a religious or mystic relationship to meditation would insist that “one-pointed concentration” is only the iceberg’s tip on the way to dissolving consciousness into oneness. Nevertheless, I take the election of “one-pointed concentration” in this experiment as a felicitous option of the authors. Why? Whenever your protocol is focussed on the potential difference between experts and novice, if you choose a type of task that is almost impossible, or bluntly unattainable, or even really impossible to the novice, you are not comparing the same type of processes but two different types. And that fact would compromise the whole experiment, as we would not be able to isolate one variable of study. Thus, by studying “one-pointed concentration”, we can understand the impact of difference of training in the physiology as a gradual difference and not as a jump to another category of mental process.

As I have a limited space here for more detailed philosophical analysis, my strategy is to shortly describe the structure of the experiment, presenting some basic philosophical comments on the experiment’s results.

## The Experiment and its Protocols: Material and Method

The experiments with functional magnetic resonance imaging (fMRI) were based initially in three groups of meditators: novice, novice with incentive and experts. They consisted of 14 experts (EMs), 16 matched age novices (NMs) and 11 novices who would have a financial incentive if they were among the top one-third in activation of attention-related areas (INs). A week before the experiment, these novices receive general instructions on how to meditate and practice concentration and two other forms of meditation 1 hour a day (20 min each). During the experiments, it was understood that splitting the experts group into two would be a better strategy to understand the results as among the experts we had considerable difference in hours of training. Thus, the group of experts was divided into LHEMs (less hours of meditation practice – mean hours: 19,000; range 10,000–24,000) and MHEMs (top four – most hours of meditation practice – mean hours: 44,000; range 37.000–52,000).

In the meditation block paradigm, the authors designed an experiment in which the participants alternated between concentration meditation and neutral resting states. This first experiment was contrasted in a cross-sectional way with another in which distracting sounds were used. The use of distracting sounds aims at testing the participants’ distractibility in the face of external stimuli. International Affective Digitized Sound was the matrix in case and the participants were exposed to 25 stimuli of 2 seconds each in a random order in terms of valences (positive, neutral and negative).

## Results and Philosophical Discussion

NMs showed less activation in attention regions of interest (ROIs) than EMs. The only exception was the thalamus. Although the difference between INMs was not very significant compared to EMs, by splitting the EMs into two groups (LHEMs and MHEMs), it was possible to trace an inverted U-shaped function in which NMs constituted the group with less activation and INM s the second lowest. LHEMs were at the top and MHEMs showed less activation than MHEMs. As the groups of LHEMs and MHEMs were age-matched and culture-matched, the main hypothesis was to credit their differences in activation to training, skill learning and plasticity. The traditional idea in Asian culture and religions that meditation starts as an effortful practice and with time turns into a more natural and less effortful process seems to be possibly represented in the results of this experiment. Achieving the same results with less cognitive effort would be a good scientific proxy of the cultural idea of a quieter mind.

Another result not much commented upon by the authors was that EMs show a more spread map of activation if we take in account the whole brain. This fact would suggest that meditation implies a form of widespread brain activation in a low and coherent level. “Quietness” would not be a “shut off” function or a literal nothingness or emptiness state, although a widespread low activation. It is especially notable that EMs show considerably less activation in discursive thought and emotional areas. This result is in accordance with the traditional idea that meditation (as a way to a “quiet mind”) is a way to dismiss language and everyday mood swings.

Distracting sounds were also used to probe concentration. EMs had less activation in areas such as posterior cingulate, precuneus and MeFG/Acc. They had also less activation in DLPFC, caudate and pulvinar. Negative correlation with hours of training was also shown in intraparietal lobule, fusiform and P. temporal. The general picture we can extract from these results is that default-mode and affective areas are negatively correlated with hours of practice, suggesting that more trained meditators were less disturbed, having less reactions to the sounds. There were positive correlations with hours of training for various areas such as insula, subthalamic, IFG and supplementary motor area. Since they were less disturbed by the sounds, it suggests that more experienced meditators would be more likely to respond in case of need of a motor action.

In the case of negatively affective sounds, the difference of EMs and the other groups was even more significant, showing less amygdala activation. These results suggest that more experienced meditators are not deeply affected or disturbed by negative afferent stimuli in accordance with the traditional idea of concentration as a mind “clean up”.

## Some Topics on Compassion Meditation

The second paper under consideration (Lutz *et al*., 2008) addresses (also using fMRI, like in Brefczynski-Lewis *et al*., 2007) a different type of meditation called compassion meditation. Compassion meditation is a technique of developing a positive feeling towards others which starts with concentrating on beloved people and after some training trying to achieve a non-referential altruistic love.

Based on fMRI and verbal self-reported intensities, it was possible to trace a correlation of more activation in ACC and insula in Good vs. Poor blocks of meditation. Increased activation in the right pSTS and TPJ among experts suggests that compassion meditation augments empathic shift of perspective and emotion sharing. The fact that the right IFG also showed greater activation in experts together with the aforementioned results would represent that experts have more sensibility to unexpected and salient behavioural stimuli. As amygdala also showed greater activation in the fMRI study, compassion meditators could be taken to be more sensitive to behaviourally salient events like the suffering of others and meta-cognitive voluntary processes such as self-controlled empathy.

## Concluding Remarks [see also Figure 1]

Meditation is a powerful form of training focus and inhibition. Attention and self-control are strictly related during meditation. Discursive thoughts and emotions are inhibited during concentration meditation. In the case of compassion meditation, emotional sensibility is increased. There are some specific points of correlation of those two types of meditation. In both cases, we have a self-regulatory process in which certain areas are inhibited. In the case of compassion meditation, instead of a spontaneous process of empathy, we would have a self-controlled activity aiming at empathy. The great difference from “one-pointed concentration” to “compassion meditation” is related to activation of emotional areas, inhibited in “one-point concentration”. Discursive thought areas are not primarily important in both forms of meditation, challenging the idea that language is essential to all forms of consciousness.

**Figure 1:**
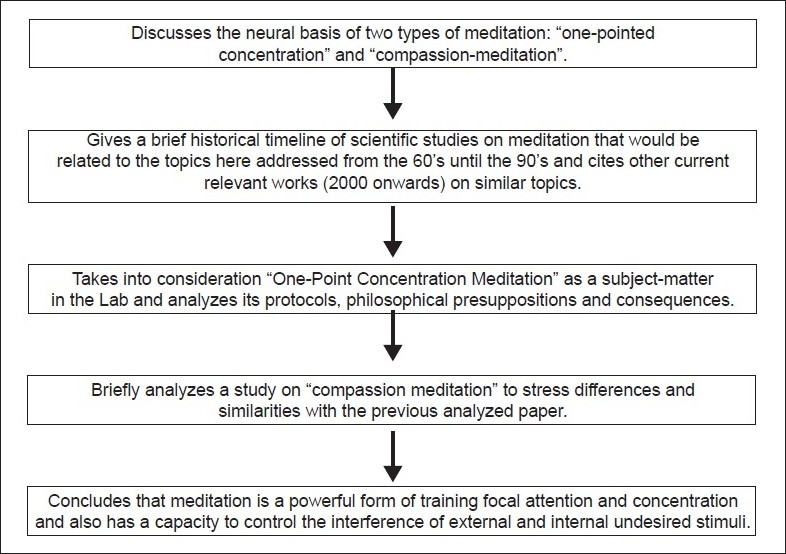
Flowchart of the Paper

### Take home message

Meditation is an important form of self-control and a healthy practice. It augments focus and attention and could be used to enhance empathy and all attentional capacities. It is worthy of practice and could lead to a better quality of lifestyle.

## Questions That This Paper Raises

1. To what extent is mental training and plasticity connected?
2. Does consciousness need a higher-order of thought, or a global pattern of coherence suffices for it?
3. How is meditation related to other forms of mental training?
4. Could meditation be used as a form of controlling automatic emotional circuitry?
5. Could meditation be used to control parasympathetic circuitry?

## About the Author

*Gabriel J. C. Mograbi is Professor of Epistemology, Philosophy of Mind and Science at the Federal University of Mato Grosso (UFMT) in Brazil. He had previous appointments with the Federal University of Rio de Janeiro (UFRJ) and the State University of Rio de Janeiro (UERJ). Gabriel Mograbi has BA, Master and PhD degrees by the Federal University of Rio de Janeiro (UFRJ). During his PhD, he was a Visiting Scholar at the University of California, Berkeley, where he worked under the supervision of John R. Searle. Gabriel Mograbi co-ordinates two research groups on epistemology of neuroscience. He has been presenting his works at the ASSC and TSC Annual meetings and was a student member of the former association. Gabriel Mograbi also has been collaborating with Professor’s Bernard Baars’ (Researcher at The Neuroscience Institute) Web course on Consciousness held by the University of Arizona as an instructor/discussion leader.*
